# Minimal Coverability Tree Construction Made Complete and Efficient

**DOI:** 10.1007/978-3-030-45231-5_13

**Published:** 2020-04-17

**Authors:** Alain Finkel, Serge Haddad, Igor Khmelnitsky

**Affiliations:** 8 ENS/CNRS, Université Paris-Saclay, Cachan, France; 9 University of Duisburg-Essen, Duisburg, Germany; 10 LSV, ENS Paris-Saclay, CNRS, Université Paris-Saclay, Cachan, France; 11 Inria, Paris, France

**Keywords:** Petri nets, Karp-Miller tree algorithm, Coverability, Minimal coverability set, Clover, Minimal coverability tree

## Abstract

Downward closures of Petri net reachability sets can be finitely represented by their set of maximal elements called the minimal coverability set or Clover. Many properties (coverability, boundedness, ...) can be decided using Clover, in a time proportional to the size of Clover. So it is crucial to design algorithms that compute it efficiently. We present a simple modification of the original but incomplete Minimal Coverability Tree algorithm (MCT), computing Clover, which makes it complete: it memorizes accelerations and fires them as ordinary transitions. Contrary to the other alternative algorithms for which no bound on the size of the required additional memory is known, we establish that the additional space of our algorithm is at most doubly exponential. Furthermore we have implemented a prototype MinCov which is already very competitive: on benchmarks it uses less space than all the other tools and its execution time is close to the one of the fastest tool.

### 1 Introduction

**Coverability and coverability set in Petri nets.** Petri nets are iconic as an infinite-state model used for verifying concurrent systems. Coverability, in Petri nets, is the most studied property for several reasons: (1) many properties like mutual exclusion, safety, control-state reachability reduce to coverability, (2) the coverability problem is EXPSPACE-complete (while reachability is non elementary), and (3) there exist efficient prototypes and numerous case studies. To solve the coverability problem, there are backward and forward algorithms. But these algorithms do not address relevant problems like the repeated coverability problem, the LTL model-checking, the boundedness problem and regularity of the traces.

However these problems are EXPSPACE-complete [4, 1] and are also decidable using the Karp-Miller tree algorithm (KMT) [11] that computes a finite tree

⋆ The work was carried out in the framework of ReLaX, UMI2000 and also supported by ANR-17-CE40-0028 project BRAVAS.

labeled by a set of *ω-markings* $C \subseteq \mathbb{N}_\omega^P$ (where $\mathbb{N}_\omega$ is the set of naturals enlarged with an upper bound $\omega$ and $P$ is the set of places) such that the reachability set and the finite set $C$ have the same downward closure in $\mathbb{N}^P$. Thus a marking $\mathbf{m}$ is coverable if there exists some $\mathbf{m}' \geq \mathbf{m}$ with $\mathbf{m}' \in C$. Hence, $C$ can be seen as *one* among all the possible finite representations of the infinite downward closure of the reachability set. This set $C$ allows, for instance, to solve multiple instances of coverability in linear time linear w.r.t. the size of $C$ avoiding to call many times a costly algorithm. Informally the KMT algorithm builds a reachability tree but, in order to ensure termination, substitutes $\omega$ to some finite components of a marking of a vertex when some marking of an ancestor is smaller.

Unfortunately $C$ may contain comparable markings while only the maximal elements are important. The set of maximal elements of $C$ can be defined independently of the KMT algorithm and was called the *minimal coverability set (MCS)* in [6] and abbreviated as the *Clover* in the more general framework of Well Structured Transition Systems (WSTS) [7].

**The minimal coverability tree algorithm.** So in [5,6] the author computes the minimal coverability set by modifying the KMT algorithm in such a way that at each step of the algorithm, the set of $\omega$-markings labelling vertices is an antichain. But this aggressive strategy, implemented by the so-called Minimal Coverability Tree algorithm (MCT), contains a subtle bug and it may compute a strict under-approximation of Clover as shown in [8,10].

**Alternative minimal coverability set algorithms.** Since the discovery of this bug, three algorithms (with variants) [10,14,13] have been designed for computing the minimal coverability set without building the full Karp-Miller tree. In [10] the authors proposed a minimal coverability set algorithm (called `CovProc`) that is not based on the Karp-Miller tree algorithm but uses a similar but restricted introduction of $\omega$'s. In [14], Reynier and Servais proposed a modification of the MCT, called the Monotone-Pruning algorithm (called `MP`), that keeps but "deactivates" vertices labeled with smaller $\omega$-markings while MCT would have deleted them. Recently in [15], the authors simplified their original proof of correctness. In [16], Valmari and Hansen proposed another algorithm (denoted below as `VH`) for constructing the minimal coverability set without deleting vertices. Their algorithm builds a graph and not a tree as usual. In [13], Piipponen and Valmari improved this algorithm by designing appropriate data structures and heuristics for exploration strategy that may significantly decrease the size of the graph.

**Our contributions.**

1. We introduce the concept of *abstraction* as an $\omega$-transition that mimics the effect of an infinite family of firing sequences of markings w.r.t. coverability. As a consequence adding abstractions to the net does not modify its coverability set. Moreover, the classical Karp-Miller *acceleration* can be formalized as an abstraction whose incidence on places is either $\omega$ or null. The set of accelerations of a net is upward closed and well-ordered. Hence there exists a finite subset of minimal accelerations and we show that the size of all minimal acceleration is bounded by a double exponential.

2. Despite the current opinion that *"The flaw is intricate and we do not see an easy way to get rid of it....Thus, from our point of view, fixing the bug of the MCT algorithm seems to be a difficult task"* [10], we have found a *simple* modification of MCT which makes it correct. It mainly consists in memorizing discovered accelerations and using them as ordinary transitions.
3. Contrary to *all* existing minimal coverability set algorithms that use an *unknown additional memory* that could be non primitive recursive, we show, by applying a recent result of Leroux [12], that the additional memory required for accelerations, is at most doubly exponential.
4. We have developed a prototype in order to also empirically evaluate the efficiency of our algorithm and the benchmarks (either from the literature or random ones) have confirmed that our algorithm requires significantly less memory than the other algorithms and is close to the fastest tool w.r.t. the execution time.

**Organization.** Section 2 introduces abstractions and accelerations and studies their properties. Section 3 presents our algorithm and establishes its correctness. Section 4 describes our tool and discusses the results of the benchmarks. We conclude and give some perspectives to this work in Section 5. One can find all the missing proofs and an illustration of the behavior of the algorithm in [9].

### 2 Covering abstractions

#### 2.1 Petri nets: reachability and covering

Here we define Petri nets differently from the usual way but in an equivalent manner. i.e. based on the backward incidence matrix **Pre** and the incidence matrix **C**. The forward incidence matrix is implicitly defined by $\mathbf{C} + \mathbf{Pre}$. Such a choice is motivated by the introduction of abstractions in section 2.2.

**Definition 1.** *A Petri net (PN) is a tuple* $\mathcal{N} = \langle P, T, \mathbf{Pre}, \mathbf{C} \rangle$ *where:*

- $P$ *is a finite set of* places*;*
- $T$ *is a finite set of* transitions*, with* $P \cap T = \emptyset$*;*
- $\mathbf{Pre} \in \mathbb{N}^{P \times T}$ *is the* backward incidence matrix*;*
- $\mathbf{C} \in \mathbb{Z}^{P \times T}$ *is the* incidence matrix *which fulfills:* *for all* $p \in P$ *and* $t \in T$*,* $\mathbf{C}(p,t) + \mathbf{Pre}(p,t) \geq 0$.

*A* marked *Petri net* $(\mathcal{N}, \mathbf{m}_0)$ *is a Petri net* $\mathcal{N}$ *equipped with an initial marking* $\mathbf{m}_0 \in \mathbb{N}^P$.

The column vector of matrix **Pre** (resp. **C**) indexed by $t \in T$ is denoted $\mathbf{Pre}(t)$ (resp. $\mathbf{C}(t)$). A transition $t \in T$ is *fireable* from a marking $\mathbf{m} \in \mathbb{N}^P$ if $\mathbf{m} \geq \mathbf{Pre}(t)$. When $t$ is fireable from $\mathbf{m}$, its *firing* leads to marking $\mathbf{m}' \stackrel{\text{def}}{=} \mathbf{m} + \mathbf{C}(t)$, denoted by $\mathbf{m} \xrightarrow{t} \mathbf{m}'$. One extends fireability and firing to a sequence $\sigma \in T^*$ by recurrence on its length. The empty sequence $\varepsilon$ is always fireable and let the marking unchanged. Let $\sigma = t\sigma'$ be a sequence with $t \in T$ and $\sigma' \in T^*$. Then $\sigma$

is fireable from $\mathbf{m}$ if $\mathbf{m} \xrightarrow{t} \mathbf{m}'$ and $\sigma'$ is fireable from $\mathbf{m}'$. The firing of $\sigma$ from $\mathbf{m}$ leads to the marking $\mathbf{m}''$ reached by $\sigma'$ from $\mathbf{m}'$. One also denotes this firing by $\mathbf{m} \xrightarrow{\sigma} \mathbf{m}''$.

**Definition 2.** *Let* $(\mathcal{N}, \mathbf{m}_0)$ *be a marked net. The* reachability set $Reach(\mathcal{N}, \mathbf{m}_0)$ *is defined by:*

$$Reach(\mathcal{N}, \mathbf{m}_0) = \{\mathbf{m} \mid \exists \sigma \in T^* \ \mathbf{m}_0 \xrightarrow{\sigma} \mathbf{m}\}$$

In order to introduce the coverability set of a Petri net, let us recall some definitions and results related to ordered sets. Let $(X, \leq)$ be an ordered set. The downward (resp. upward) *closure* of a subset $E \subseteq X$ is denoted by $\downarrow E$ (resp. $\uparrow E$) and defined by:

$$\downarrow E = \{x \in X \mid \exists y \in E \ y \geq x\} \qquad (\text{resp. } \uparrow E = \{x \in X \mid \exists y \in E \ y \leq x\})$$

A subset $E \subseteq X$ is downward (resp. upward) *closed* if $E = \downarrow E$ (resp. $E = \uparrow E$).

An *antichain* $E$ is a set which fulfills: $\forall x \neq y \in E \ \neg(x \leq y \vee y \leq x)$. $X$ is said *FAC* (for Finite AntiChains) if all its antichains are finite. A non empty set $E \subseteq X$ is *directed* if for all $x, y \in E$ there exists $z \in E$ such that $x \leq z$ and $y \leq z$. An *ideal* is a set which is downward closed and directed. There exists an equivalent characterization of FAC sets which provides a finite description of any downward closed set: a set is FAC if and only if every downward closed set admits a finite decomposition in ideals (a proof of this well-known result can be found in [3]).

$X$ is *well founded* if all its (strictly) decreasing sequences are finite. $X$ is *well ordered* if it is FAC and well founded. There are many equivalent characterizations of well order. For instance, a set $X$ is well ordered if and only if for all sequence $(x_n)_{n \in \mathbb{N}}$ in $X$, there exists a non decreasing infinite subsequence. This characterization allows to design algorithms that computes trees whose finiteness is ensured by well order. Let us recall that $(\mathbb{N}, \leq)$ and $(\mathbb{N}^P, \leq)$ are well ordered sets.

We are now ready to introduce the *cover* (also called the coverability set) of a net and to state some of its properties.

**Definition 3.** *Let* $(\mathcal{N}, \mathbf{m}_0)$ *be a marked Petri net.* $Cover(\mathcal{N}, \mathbf{m}_0)$*, its* coverability set*, is defined by:*

$$Cover(\mathcal{N}, \mathbf{m}_0) = \downarrow Reach(\mathcal{N}, \mathbf{m}_0)$$

Since the coverability set is downward closed and $\mathbb{N}^P$ is FAC, it admits a finite decomposition in ideals. The ideals of $\mathbb{N}^P$ can be defined in an elegant way as follows. One first extends the sets of naturals and integers: $\mathbb{N}_\omega = \mathbb{N} \cup \{\omega\}$ et $\mathbb{Z}_\omega = \mathbb{Z} \cup \{\omega\}$. Then one extends the order relation and the addition to $\mathbb{Z}_\omega$: for all $n \in \mathbb{Z}$, $\omega > n$ and for all $n \in \mathbb{Z}_\omega$, $n + \omega = \omega + n = \omega$. $\mathbb{N}_\omega^P$ is also a well ordered set and its members are called *$\omega$-markings*. There is a one-to-one mapping between ideals of $\mathbb{N}^P$ and $\omega$-markings. Let $\mathbf{m} \in \mathbb{N}_\omega^P$. Define $[\![\mathbf{m}]\!]$ by:

$$[\![\mathbf{m}]\!] = \{\mathbf{m}' \in \mathbb{N}^P \mid \mathbf{m}' \leq \mathbf{m}\}$$

$[\![\mathbf{m}]\!]$ is an ideal of $\mathbb{N}^P$ (and all ideal can be defined in such a way). Let $\Omega$ be a set of $\omega$-markings, $[\![\Omega]\!]$ denotes the set $\bigcup_{\mathbf{m}\in\Omega}[\![\mathbf{m}]\!]$. Due to the above properties, there exists a unique finite set with minimal size $Clover(\mathcal{N},\mathbf{m}_0)\subseteq\mathbb{N}_\omega^p$ such that:

$$Cover(\mathcal{N},\mathbf{m}_0)=[\![Clover(\mathcal{N},\mathbf{m}_0)]\!]$$

A more general result can be found in [3] for well structured transition systems.

*Example 1.* The marked net of Figure 1 is unbounded. Its Clover is the following set:

$$\{p_i, p_{bk}+p_m, p_l+p_m+\omega p_{ba}, p_l+p_{bk}+\omega p_{ba}+\omega p_c\}$$

For instance, the marking $p_l+p_{bk}+\alpha p_{ba}+\beta p_c$ is reached thus covered by sequence $t_1 t_5^{\alpha+\beta} t_6^{\beta}$.

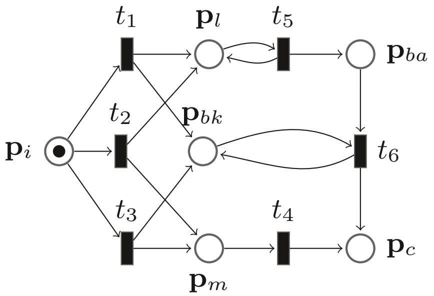

**Fig. 1.** An unbounded Petri net

### 2.2 Abstraction and acceleration

In order to introduce abstractions and accelerations, we generalize the transitions to allow the capability to mark a place with $\omega$ tokens.

**Definition 4.** *Let $P$ be a set of places. An $\omega$-transition* $\mathbf{a}$ *is defined by:*

- $\mathbf{Pre}(\mathbf{a})\in\mathbb{N}_\omega^P$ *its* backward incidence*;*
- $\mathbf{C}(\mathbf{a})\in\mathbb{Z}_\omega^P$ *its* incidence *with* $\mathbf{Pre}(\mathbf{a})+\mathbf{C}(\mathbf{a})\geq 0$.

For sake of homogeneity, one denotes $\mathbf{Pre}(\mathbf{a})(p)$ (resp. $\mathbf{C}(\mathbf{a})(p)$) by $\mathbf{Pre}(p,\mathbf{a})$ (resp. $\mathbf{C}(p,\mathbf{a})$). An $\omega$-transition $\mathbf{a}$ is fireable from an $\omega$-marking $\mathbf{m}\in\mathbb{N}_\omega^P$ if $\mathbf{m}\geq\mathbf{Pre}(\mathbf{a})$. When $\mathbf{a}$ is fireable from $\mathbf{m}$, its firing leads to the $\omega$-marking $\mathbf{m}'\stackrel{\text{def}}{=}\mathbf{m}+\mathbf{C}(\mathbf{a})$, denoted as previously $\mathbf{m}\xrightarrow{\mathbf{a}}\mathbf{m}'$. One observes that if $\mathbf{Pre}(p,\mathbf{a})=\omega$ then for all values of $\mathbf{C}(p,\mathbf{a})$, $\mathbf{m}'(\mathbf{a})=\omega$. So without loss of generality, one assumes that for all $\omega$-transition $\mathbf{a}$, $\mathbf{Pre}(p,\mathbf{a})=\omega$ implies $\mathbf{C}(p,\mathbf{a})=\omega$.

In order to define abstractions, we first define the incidences of a sequence $\sigma$ of $\omega$-transitions by recurrence on its length. As previously, we denote $\mathbf{Pre}(p,\sigma)\stackrel{\text{def}}{=}$

$\mathbf{Pre}(\sigma)(p)$ and $\mathbf{C}(p,\sigma) \stackrel{\text{def}}{=} \mathbf{C}(\sigma)(p)$. The base case corresponds to the definition of an $\omega$-transition. Let $\sigma = t\sigma'$, with $t$ an $\omega$-transition and $\sigma'$ a sequence of $\omega$-transitions, then:

- $\mathbf{C}(\sigma) = \mathbf{C}(t) + \mathbf{C}(\sigma')$;
- for all $p \in P$
  - if $\mathbf{C}(p,t) = \omega$ then $\mathbf{Pre}(p,\sigma) = \mathbf{Pre}(p,t)$;
  - else $\mathbf{Pre}(p,\sigma) = \max(\mathbf{Pre}(p,t), \mathbf{Pre}(p,\sigma') - \mathbf{C}(p,t))$.

One checks by recurrence that $\sigma$ is firable from $\mathbf{m}$ if and only if $\mathbf{m} \geq \mathbf{Pre}(\sigma)$ and in this case, $\mathbf{m} \xrightarrow{\sigma} \mathbf{m} + \mathbf{C}(\sigma)$.

An *abstraction* of a net is an $\omega$-transition which concisely expresses the behaviour of the net w.r.t. covering (see Proposition 1). One will observe that a transition $t$ of a net is by construction (with $\sigma_n = t$ for all $n$) an abstraction.

**Definition 5.** *Let $\mathcal{N} = \langle P, T, \mathbf{Pre}, \mathbf{C}\rangle$ be a Petri net and $\mathbf{a}$ be an $\omega$-transition. $\mathbf{a}$ is an* abstraction *if for all $n \geq 0$, there exists $\sigma_n \in T^*$ such that for all $p \in P$ with $\mathbf{Pre}(p,\mathbf{a}) \in \mathbb{N}$:*

1. *$\mathbf{Pre}(p,\sigma_n) \leq \mathbf{Pre}(p,\mathbf{a})$;*
2. *If $\mathbf{C}(p,\mathbf{a}) \in \mathbb{Z}$ then $\mathbf{C}(p,\sigma_n) \geq \mathbf{C}(p,\mathbf{a})$;*
3. *If $\mathbf{C}(p,\mathbf{a}) = \omega$ then $\mathbf{C}(p,\sigma_n) \geq n$.*

The following proposition justifies the interest of abstractions.

**Proposition 1.** *Let $(\mathcal{N}, \mathbf{m}_0)$ be a marked Petri net, $\mathbf{a}$ be an abstraction and $\mathbf{m}$ be an $\omega$-marking such that: $[\![\mathbf{m}]\!] \subseteq Cover(\mathcal{N}, \mathbf{m}_0)$ and $\mathbf{m} \xrightarrow{\mathbf{a}} \mathbf{m}'$. Then $[\![\mathbf{m}']\!] \subseteq Cover(\mathcal{N}, \mathbf{m}_0)$.*

**Proof.** Pick some $\mathbf{m}^* \in [\![\mathbf{m}']\!]$. Denote $n = \max(\mathbf{m}^*(p) \mid \mathbf{m}'(p) = \omega)$ and $\ell = \max(\mathbf{Pre}(p,\sigma_n), n - \mathbf{C}(p,\sigma_n) \mid \mathbf{m}(p) = \omega)$. Let us define $\mathbf{m}^\sharp \in [\![\mathbf{m}]\!]$ by:

- If $\mathbf{m}(p) < \omega$ then $\mathbf{m}^\sharp(p) = \mathbf{m}(p)$;
- Else $\mathbf{m}^\sharp(p) = \ell$.

Let us check that $\sigma_n$ is fireable from $\mathbf{m}^\sharp$. Let $p \in P$,

- If $\mathbf{m}(p) < \omega$ then $\mathbf{m}^\sharp(p) = \mathbf{m}(p) \geq \mathbf{Pre}(p,\mathbf{a}) \geq \mathbf{Pre}(p,\sigma_n)$;
- Else $\mathbf{m}^\sharp(p) = \ell \geq \mathbf{Pre}(p,\sigma_n)$.

Let us show that $\mathbf{m}^\sharp + \mathbf{C}(\sigma_n) \geq \mathbf{m}^*$. Let $p \in P$,

- If $\mathbf{m}(p) < \omega$ and $\mathbf{C}(p,\mathbf{a}) < \omega$ then $\mathbf{m}^\sharp(p) + \mathbf{C}(p,\sigma_n) \geq \mathbf{m}(p) + \mathbf{C}(p,\mathbf{a}) = \mathbf{m}'(p) \geq \mathbf{m}^*(p)$;
- If $\mathbf{m}(p) < \omega$ and $\mathbf{C}(p,\mathbf{a}) = \omega$ then $\mathbf{m}^\sharp(p) + \mathbf{C}(p,\sigma_n) \geq \mathbf{C}(p,\sigma_n) \geq n \geq \mathbf{m}^*(p)$ ;
- If $\mathbf{m}(p) = \omega$ then $\mathbf{m}^\sharp(p) + \mathbf{C}(p,\sigma_n) \geq n - \mathbf{C}(p,\sigma_n) + \mathbf{C}(p,\sigma_n) = n \geq \mathbf{m}^*(p)$.

∎

An easy way to build new abstractions consists in concatenating them.

**Proposition 2.** *Let $\mathcal{N} = \langle P, T, \mathbf{Pre}, \mathbf{C}\rangle$ be a Petri net and $\sigma$ be a sequence of abstractions. Then the $\omega$-transition* $\mathbf{a}$ *defined by* $\mathbf{Pre}(\mathbf{a}) = \mathbf{Pre}(\sigma)$ *and* $\mathbf{C}(\mathbf{a}) = \mathbf{C}(\sigma)$ *is an abstraction.*

We now introduce the underlying concept of the Karp and Miller construction.

**Definition 6.** *Let $\mathcal{N} = \langle P, T, \mathbf{Pre}, \mathbf{C}\rangle$ be a Petri net. One says that* $\mathbf{a}$ *is an* acceleration *if* $\mathbf{a}$ *is an abstraction such that* $\mathbf{C}(\mathbf{a}) \in \{0, \omega\}^P$.

The following proposition provides a way to get an acceleration from an arbitrary abstraction.

**Proposition 3.** *Let $\mathcal{N} = \langle P, T, \mathbf{Pre}, \mathbf{C}\rangle$ be a Petri net and* $\mathbf{a}$ *be an abstraction. Define* $\mathbf{a}'$ *an $\omega$-transition as follows. For all $p \in P$:*

– *If* $\mathbf{C}(p, \mathbf{a}) < 0$ *then* $\mathbf{Pre}(p, \mathbf{a}') = \mathbf{C}(p, \mathbf{a}') = \omega$*;*
– *If* $\mathbf{C}(p, \mathbf{a}) = 0$ *then* $\mathbf{Pre}(p, \mathbf{a}') = \mathbf{Pre}(p, \mathbf{a})$ *and* $\mathbf{C}(p, \mathbf{a}') = 0$*;*
– *If* $\mathbf{C}(p, \mathbf{a}) > 0$ *then* $\mathbf{Pre}(p, \mathbf{a}') = \mathbf{Pre}(p, \mathbf{a})$ *and* $\mathbf{C}(p, \mathbf{a}') = \omega$*.*

*Then* $\mathbf{a}'$ *is an acceleration.*

Let us study more deeply the set of accelerations. First we equip the set of $\omega$-transitions with a"natural" order w.r.t. covering.

**Definition 7.** *Let $P$ be a set of places and two $\omega$-transitions* $\mathbf{a}$ *and* $\mathbf{a}'$*.*

$$\mathbf{a} \leq \mathbf{a}' \textit{ if and only if } \mathbf{Pre}(\mathbf{a}) \leq \mathbf{Pre}(\mathbf{a}') \wedge \mathbf{C}(\mathbf{a}) \geq \mathbf{C}(\mathbf{a}')$$

In other words, $\mathbf{a} \leq \mathbf{a}'$ if given any $\omega$-marking $\mathbf{m}$, if $\mathbf{a}'$ is fireable from $\mathbf{m}$ then $\mathbf{a}$ is also fireable and its firing leads to a marking greater or equal that the one reached by the firing of $\mathbf{a}'$.

**Proposition 4.** *Let $\mathcal{N}$ be a Petri net. Then the set of abstractions of $\mathcal{N}$ is upward closed. Similarly, the set of accelerations is upward closed in the set of $\omega$-transitions whose incidence belongs to $\{0, \omega\}^P$.*

**Proposition 5.** *The set of accelerations of a Petri net is well ordered.*

**Proof.** The set of accelerations is a subset of $\mathbb{N}^P \times \{0, \omega\}^P$ (where $P$ is the set of places) with the order obtained by iterating cartesian products of sets $(\mathbb{N}, \leq)$ and $(\{0, \omega\}, \geq)$. These sets are well ordered and the cartesian product preserves this property. So we are done. ■

Since the set of accelerations is well ordered and it is upward closed, it is equal to the upward closure of the finite set of *minimal* accelerations. Let us study the size of a minimal acceleration. Given some Petri net, one denotes $d = |P|$ and $e = \max_{p,t}(\max(\mathbf{Pre}(p, t), \mathbf{Pre}(p, t) + \mathbf{C}(p, t)))$.

We are going to use the following result of Jérôme Leroux (published on HAL in June 2019) which provides a bound for the lengths of shortest sequences between two markings $\mathbf{m}_1$ and $\mathbf{m}_2$ mutually reachable.

**Theorem 1.** *(Theorem 2, [12]) Let $\mathcal{N}$ be a Petri net, $\mathbf{m}_1, \mathbf{m}_2$ be markings, $\sigma_1, \sigma_2$ be sequences of transitions such that $\mathbf{m}_1 \xrightarrow{\sigma_1} \mathbf{m}_2 \xrightarrow{\sigma_2} \mathbf{m}_1$. Then there exist $\sigma'_1, \sigma'_2$ such that $\mathbf{m}_1 \xrightarrow{\sigma'_1} \mathbf{m}_2 \xrightarrow{\sigma'_2} \mathbf{m}_1$ fulfilling:*

$$|\sigma'_1\sigma'_2| \leq ||\mathbf{m}_1 - \mathbf{m}_2||_\infty (3de)^{(d+1)^{2d+4}}$$

One deduces an upper bound on the size of minimal accelerations.
Let $\mathbf{v} \in \mathbb{N}_\omega^P$. One denotes $||\mathbf{v}||_\infty = \max(\mathbf{v}(p) \mid \mathbf{v}(p) \in \mathbb{N})$.

**Proposition 6.** *Let $\mathcal{N}$ be a Petri net and $\mathbf{a}$ be a minimal acceleration. Then $||\mathbf{Pre}(\mathbf{a})||_\infty \leq e(3de)^{(d+1)^{2d+4}}$.*

**Proof.** Let us consider the net $\mathcal{N}' = \langle P', T', \mathbf{Pre}', \mathbf{C}' \rangle$ obtained from $\mathcal{N}$ by deleting the set of places $\{p \mid \mathbf{Pre}(p, \mathbf{a}) = \omega\}$ and adding the set of transitions $T_1 = \{t_p \mid p \in P'\}$ with $\mathbf{Pre}(t_p) = p$ et $\mathbf{C}(t_p) = -p$. Observe that $d' \leq d$ and $e' = e$.
One denotes $P_1 = \{p \mid \mathbf{Pre}(p, \mathbf{a}) < \omega = \mathbf{C}(p, \mathbf{a})\}$. One introduces $\mathbf{m}_1$ the marking obtained by restricting $\mathbf{Pre}(\mathbf{a})$ to $P'$ and $\mathbf{m}_2 = \mathbf{m}_1 + \sum_{p \in P_1} p$.
Let $\{\sigma_n\}_{n\in\mathbb{N}}$ be a family of sequences associated with $\mathbf{a}$. Let $n^* = ||\mathbf{Pre}(\mathbf{a})||_\infty + 1$. Then $\sigma_{n^*}$ is fireable in $\mathcal{N}'$ from $\mathbf{m}_1$ and its firing leads to a marking that covers $\mathbf{m}_2$. By concatenating some occurrences of transitions of $T_1$, one gets a firing sequence in $\mathcal{N}'$ $\mathbf{m}_1 \xrightarrow{\sigma_1} \mathbf{m}_2$. Using the same process, one gets a firing sequence $\mathbf{m}_2 \xrightarrow{\sigma_2} \mathbf{m}_1$.
Let us apply Theorem 1. There exists a sequence $\sigma'_1$ with $\mathbf{m}_1 \xrightarrow{\sigma'_1} \mathbf{m}_2$ and $|\sigma'_1| \leq (3de)^{(d+1)^{2d+4}}$ since $||\mathbf{m}_1 - \mathbf{m}_2||_\infty = 1$. By deleting the transitions of $T_1$ occurring in $\sigma'_1$, one gets a sequence $\sigma''_1 \in T^*$ such that $\mathbf{m}_1 \xrightarrow{\sigma''_1} \mathbf{m}'_2 \geq \mathbf{m}_2$ with $|\sigma''_1| \leq (3de)^{(d+1)^{2d+4}}$.
The $\omega$-transition $\mathbf{a}'$, defined by $\mathbf{Pre}(p, \mathbf{a}') = \mathbf{Pre}(p, \sigma''_1)$ for all $p \in P'$, $\mathbf{Pre}(p, \mathbf{a}') = \omega$ for all $p \in P \setminus P'$ and $\mathbf{C}(\mathbf{a}') = \mathbf{C}(\mathbf{a})$, is an acceleration whose associated family is $\{\sigma''^n_1\}_{n\in\mathbb{N}}$. By definition of $\mathbf{m}_1$, $\mathbf{a}' \leq \mathbf{a}$. Since $\mathbf{a}$ is minimal, $\mathbf{a}' = \mathbf{a}$. Observing that $|\sigma''_1| \leq (3de)^{(d+1)^{2d+4}}$, one gets $||\mathbf{Pre}(\mathbf{a})||_\infty = ||\mathbf{Pre}(\mathbf{a}')||_\infty \leq e(3de)^{(d+1)^{2d+4}}$.

■

Thus given any acceleration, one can easily obtain a smaller acceleration whose (representation) size is exponential.

**Proposition 7.** *Let $\mathcal{N}$ be a Petri net and $\mathbf{a}$ be an acceleration. Then the $\omega$-transition $trunc(\mathbf{a})$ defined by:*

- $\mathbf{C}(trunc(\mathbf{a})) = \mathbf{C}(\mathbf{a})$;
- *for all $p$ such that $\mathbf{Pre}(p, \mathbf{a}) \neq \omega$,* $\mathbf{Pre}(p, trunc(\mathbf{a})) = \min(\mathbf{Pre}(p, \mathbf{a}), e(3de)^{(d+1)^{2d+4}})$ ;
- *for all $p$ such that $\mathbf{Pre}(p, \mathbf{a}) = \omega$, $\mathbf{Pre}(p, trunc(\mathbf{a})) = \omega$.*

*is an acceleration.*

**Proof.** Let $\mathbf{a}' \leq \mathbf{a}$, be a minimal acceleration. For all $p$ such that $\mathbf{Pre}(p, \mathbf{a}) \neq \omega$, $Pre(p, \mathbf{a}') \leq e(3de)^{(d+1)^{2d+4}}$. So $\mathbf{a}' \leq trunc(\mathbf{a})$. Since the set of accelerations is upward closed, one gets that $trunc(\mathbf{a})$ is an acceleration. ■

## 3 A coverability tree algorithm

### 3.1 Specification and illustration

As discussed in the introduction, to compute the clover of a Petri net, most algorithms build coverability trees (or graphs), which are variants of the Karp and Miller tree with the aim of reducing the peak memory during the execution. The seminal algorithm [6] is characterized by a main difference with the KMT construction: when finding that the marking associated with the current vertex strictly covers the marking of another vertex, it deletes the subtree issued from this vertex, and when the current vertex belonged to the removed subtree it substitutes it to the root of the deleted subtree. This operation drastically reduces the peak memory but as shown in [8] entails incompleteness of the algorithm.

Like the previous algorithms that ensure completeness with deletions, our algorithm also needs additional memory. However unlike the other algorithms, it memorizes accelerations instead of $\omega$-markings. This approach has two advantages. First, we are able to exhibit a theoretical upper bound on the additional memory which is doubly exponential, while the other algorithms do not have such a bound. Furthermore, accelerations are reused in the construction and thus may even shorten the execution time and peak space w.r.t. the algorithm in [6].

Before we delve into a high level description of this algorithm, let us present some of the variables, functions, and definitions used by the algorithm. Algorithm 1, denoted from now on as `MinCov` takes as an input a marked net $(\mathcal{N}, \mathbf{m}_0)$ and constructs a directed labeled tree $CT = (V, E, \lambda, \delta)$, and a set $\mathsf{Acc}$ of $\omega$-transitions (which by Lemma 2 are accelerations). Each $v \in V$ is labeled by an $\omega$-marking, $\lambda(v) \in \mathbb{N}_\omega^P$. Since $CT$ is a directed tree, every vertex $v \in V$, has a predecessor (except the root $r$) denoted by $prd(v)$ and a set of descendants denoted by $Des(v)$. By convention, $prd(r) = r$. Each edge $e \in E$ is labeled by a firing sequence $\delta(e) \in T_o \cdot \mathsf{Acc}^*$, consisting of an ordinary transition followed by a sequence of accelerations (which by Lemma 1 fulfills $\lambda(prd(v)) \xrightarrow{\delta(prd(v),v)} \lambda(v)$). In addition, again by Lemma 1, $\mathbf{m}_0 \xrightarrow{\delta(r,r)} \lambda(r)$. Let $\gamma = e_1 e_2 \dots e_k \in E^*$ be a path in the tree, we denote by $\delta(\gamma) := \delta(e_1)\delta(e_2)\dots\delta(e_k) \in (T \cup \mathsf{Acc})^*$. The subset $\mathsf{Front} \subset V$ is the set of vertices 'to be processed'.

`MinCov` may call function $\texttt{Delete}(v)$ that removes from $V$ a leaf $v$ of $CT$ and function $\texttt{Prune}(v)$ that removes from $V$ all descendants of $v \in V$ except $v$ itself as illustrated in the following figure:

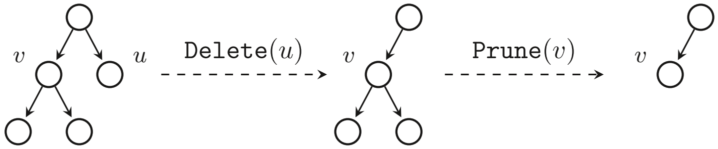

First `MinCov` does some initializations, and sets the tree $CT$ to be a single vertex $r$ with marking $\lambda(r) = \mathbf{m}_0$ and $\mathsf{Front} = \{r\}$. Afterwards the main loop builds the tree, where each iteration consists in processing some vertex in Front as follows.

MinCov picks a vertex $u \in \mathsf{Front}$ (line 3). From $\lambda(u)$, MinCov fires a sequence $\sigma \in Acc^*$ reaching some $\mathbf{m}_u$ that maximizes the number of $\omega$ produced, i.e. $|\{p \in P \mid \lambda(u)(p) \neq \omega \wedge \mathbf{m}_u(p) = \omega\}|$. Thus in $\sigma$, no acceleration occurs twice and its length is bounded by $|P|$. Then MinCov updates $\lambda(u)$ with $\mathbf{m}_u$ (line 5) and the label of the edge incoming to $u$ by concatenating $\sigma$. Afterwards it performs one of the following actions according to the marking $\lambda(u)$:

- **Cleaning** (line 7): If there exists $u' \in V \setminus \mathsf{Front}$ with $\lambda(u') \geq \lambda(u)$. The vertex $u$ is redundant and MinCov calls `Delete`$(u)$
- **Accelerating** (lines 8-16): If there exists $u'$, an ancestor of $u$ with $\lambda(u') < \lambda(u)$ then an acceleration can be computed. The acceleration $\mathbf{a}$ is deduced from the firing sequence labeling the path from $u'$ to $u$. MinCov inserts $\mathbf{a}$ into Acc, calls `Prune`$(u')$ and pushes back $u'$ in Front.
- **Exploring** (lines 18 - 25): Otherwise MinCov calls `Prune`$(u')$ followed by `Delete`$(u')$ for all $u' \in V$ with $\lambda(u') < \lambda(u)$ since they are redundant. Afterwards, it removes $u$ from Front and for all fireable transition $t \in T$ from $\lambda(u)$, it creates a new child for $u$ in $CT$ and inserts it into Front.

For a detailed example of a run of the algorithm see Example 2 in [9].

#### 3.2 Correctness Proof

We now establish the correctness of Algorithm 1 by proving the following properties (where for all $W \subseteq V$, $\lambda(W)$ denotes $\bigcup_{v \in W} \lambda(v)$):

- its termination;
- the incomparability of $\omega$-markings associated with vertices in $V$: $\lambda(V)$ is an antichain;
- its consistency: $[\![\lambda(V)]\!] \subseteq Cover(\mathcal{N}, \mathbf{m}_0)$;
- its completeness: $Cover(\mathcal{N}, \mathbf{m}_0) \subseteq [\![\lambda(V)]\!]$.

We get termination by using the well order of $\mathbb{N}_\omega^P$ and Koenig Lemma.

**Proposition 8.** *MinCov terminates.*

**Proof.** Consider the following variation of the algorithm.

Instead of deleting the current vertex when its marking is smaller or equal than the marking of a vertex, one marks it as ‘cut’ and extract it from Front.

Instead of cutting a subtree when the marking of the current vertex $v$ is greater than the marking of a vertex which is not an ancestor of $v$, one marks them as ‘cut’ and extract from Front those who are inside.

Instead of cutting a subtree when the marking of the current vertex $v$ is greater than the marking of a vertex which is an ancestor of $v$, say $v^*$, one marks those on the path from $v^*$ to $v$ (except $v$) as ‘accelerated’, one marks the other vertices

**Algorithm 1:** Computing the minimal coverability set

`MinCov`$(\mathcal{N}, \mathbf{m}_0)$
**Input:** A marked Petri net $(\mathcal{N}, \mathbf{m}_0)$
**Data:** $V$ set of vertices; $E \subseteq V \times V$; $\mathsf{Front} \subseteq V$; $\lambda : V \to \mathbb{N}^p_\omega$; $\delta : E \to T_o\mathsf{Acc}^*$; $CT = (V, E, \lambda, \delta)$ a labeled tree; $\mathsf{Acc}$ a set of $\omega$-transitions;
**Output:** A labeled tree $CT = (V, E, \lambda, \delta)$

$V \leftarrow \{r\}$; $E \leftarrow \emptyset$; $\mathsf{Front} \leftarrow \{r\}$; $\lambda(r) \leftarrow \mathbf{m}_0$; $\mathsf{Acc} \leftarrow \emptyset$; $\delta(r, r) \leftarrow \varepsilon$
**while** $Front \neq \emptyset$ **do**
    `Select` $u \in \mathsf{Front}$
    Let $\sigma \in \mathsf{Acc}^*$ a maximal fireable sequence of accelerations from $\lambda(u)$
    `// Maximal w.r.t. the number of` $\omega$`'s produced`
    $\lambda(u) \leftarrow \lambda(u) + \mathbf{C}(\sigma)$
    $\delta((prd(u), u)) \leftarrow \delta((prd(u), u)) \cdot \sigma$
    **if** $\exists u' \in V \setminus \mathsf{Front}$ $s.t.$ $\lambda(u') \geq \lambda(u)$ **then** `Delete`$(u)$ `//` $\lambda(u)$ `is covered`
    **else if** $\exists u' \in \mathsf{Anc}(V)$ $s.t.$ $\lambda(u) > \lambda(u')$ **then**
        `// An acceleration was found between` $u$ `and one of` $u$`'s ancestors`
        Let $\gamma \in E^*$ the path from $u'$ to $u$ in $CT$
        $\mathbf{a} \leftarrow$ `NewAcceleration`$()$
        **foreach** $p \in P$ **do**
            **if** $\mathbf{C}(p, \delta(\gamma)) < 0$ **then** $\mathbf{Pre}(p, \mathbf{a}) \leftarrow \omega$; $\mathbf{C}(p, \mathbf{a}) \leftarrow \omega$
            **if** $\mathbf{C}(p, \delta(\gamma)) = 0$ **then** $\mathbf{Pre}(p, \mathbf{a}) \leftarrow \mathbf{Pre}(p, \delta(\gamma))$; $\mathbf{C}(p, \mathbf{a}) \leftarrow 0$
            **if** $\mathbf{C}(p, \delta(\gamma)) > 0$ **then** $\mathbf{Pre}(p, \mathbf{a}) \leftarrow \mathbf{Pre}(p, \delta(\gamma))$; $\mathbf{C}(p, \mathbf{a}) \leftarrow \omega$
        **end**
        $\mathbf{a} \leftarrow trunc(\mathbf{a})$; $\mathsf{Acc} \leftarrow \mathsf{Acc} \cup \{\mathbf{a}\}$; `Prune`$(u')$; $\mathsf{Front} = \mathsf{Front} \cup \{u'\}$ ;
    **else**
        **for** $u' \in V$ **do**
            `// Remove vertices labeled by markings covered by` $\lambda(u)$
            **if** $\lambda(u') < \lambda(u)$ **then** `Prune`$(u')$; `Delete`$(u')$
        **end**
        $\mathsf{Front} \leftarrow \mathsf{Front} \setminus \{u\}$
        **foreach** $t \in T \wedge \lambda(u) \geq \mathbf{Pre}(t)$ **do**
            `// Add the children of` $u$
            $u' \leftarrow$ `NewNode`$()$; $V \leftarrow V \cup \{u'\}$; $\mathsf{Front} \leftarrow \mathsf{Front} \cup \{u'\})$; $E \leftarrow E \cup \{(u, u')\}$
            $\lambda(u') \leftarrow \lambda(u) + \mathbf{C}(t)$; $\delta((u, u')) \leftarrow t$
        **end**
    **end**
**end**
**return** $CT$

of the subtree as 'cut' and inserts $v$ again in Front with the marking of $v^*$. All the markings of the subtree in Front are extracted from it.

All the vertices marked as 'cut' or 'accelerated' are ignored for comparisons and discovering accelerations. This alternative algorithm behaves as the original one except that the size of the tree never decreases and so if the algorithm does not terminate the tree is infinite. Since this tree is finitely branching, due to Koenig Lemma it contains an infinite path. On this infinite path, no vertex can be marked as 'cut' since it would belong to a finite subtree. Observe that the marking labelling the vertex following an accelerated subpath has at least one more $\omega$ than the marking of the first vertex of this subpath. So there is an infinite subpath with unmarked vertices in $V$. But $\mathbb{N}_\omega^P$ is well-ordered, so there should be two vertices $v$ and $v'$, where $v'$ is a descendant of $v$ with $\lambda(v') \geq \lambda(v)$, which contradicts the behaviour of the algorithm.

■

Since we are going to use recurrence on the number of iterations of the main loop of Algorithm 1, we introduce the following notations: $CT_n = (V_n, E_n, \lambda_n, \delta_n)$, $\mathsf{Front}_n$, and $\mathsf{Acc}_n$ are the the values of variables $CT$, Front, and Acc at line 2 when $n$ iterations have been executed.

**Proposition 9.** *For all $n \in \mathbb{N}$, $\lambda(V_n \setminus \mathsf{Front}_n)$ is an antichain. Thus on termination, $\lambda(V)$ is an antichain.*

**Proof.** Let us introduce $V' := V \setminus \mathsf{Front}$ and $V'_n := V_n \setminus \mathsf{Front}_n$. We are going to prove by induction on the number $n$ of iterations of the while-loop that $V'_n$ is an antichain. `MinCov` initializes variables $V$ and Front at line 1. So $V_0 = \{r\}$ and $\mathsf{Front}_0 = \{r\}$, therefore $V'_0 = V_0 \setminus \mathsf{Front}_0 = \emptyset$ is an antichain.
Assume that $V'_n = V_n \setminus \mathsf{Front}_n$ is an antichain. Modifying $V'_n$ can be done by *adding* or *removing* vertices from $V_n$ and *removing* vertices from $\mathsf{Front}_n$ while keeping them in $V_n$. The actions that `MinCov` may perform in order to modify the sets $V$ and Front are: `Delete` (lines 7 and 19), `Prune` (lines 16 and 19), adding vertices to $V$ (line 23), adding vertices to Front (lines 16 and 23), and removing vertices from Front (line 21).
• Both `Delete` and `Prune` do not add new vertices to $V'$. Thus the antichain feature is preserved.
• `MinCov` may add vertices to $V$ only at line 23 where it simultaneously adds them to Front and therefore does not add new vertices to $V'$. Thus the antichain feature is preserved.
• Adding vertices to Front may only remove vertices from $V'_n$. Thus the antichain feature is preserved.
• `MinCov` can only add a vertex to $V'$ when it removes it from Front while keeping it in $V$. This is done only at line 21. There the only vertex `MinCov` may remove (line 21) is the working vertex $u$. However if (in the iteration) `MinCov` reaches line 21 then it did not reach line 7 hence, (1) all markings of $\lambda(V'_n) \subseteq \lambda(V_n)$ are either smaller or incomparable to $\lambda_{n+1}(u)$. Moreover, `MinCov` has also reached line 18-20, where (2) it performs `Delete` on all vertices $u' \in V'_n \subseteq V_n$ with $\lambda_n(u') < \lambda_{n+1}(u)$. Let us denote by $V''_n \subseteq V'_n$ the set $V'$ at the end of line

20. Due to (1) and (2), marking $\lambda_{n+1}(u)$ is incomparable to any marking in $\lambda_{n+1}(V''_n)$. Since $V''_n \subseteq V'_n$, $\lambda_{n+1}(V''_n)$ is an antichain. Combining this fact with the incomparability between $\lambda_{n+1}(u)$ and any marking in $\lambda_{n+1}(V''_n)$, we conclude that the set $\lambda_{n+1}(V'_{n+1}) = \lambda_{n+1}(V''_n) \cup \{\lambda_{n+1}(u)\}$ is an antichain.

■

In order to establish consistency, we prove that the labelling of vertices and edges is compatible with the firing rule and that *Acc* is a set of accelerations.

**Lemma 1.** *For all $n \in \mathbb{N}$, for all $u \in V_n \setminus \{r\}$, $\lambda_n(prd(u)) \xrightarrow{\delta(prd(u),u)} \lambda_n(u)$ and $\mathbf{m}_0 \xrightarrow{\delta(r,r)} \lambda_n(r)$.*

**Proof.** Let us prove by induction on the number $n$ of iterations of the main loop that for all $v \in V_n$, the assertions of the lemma hold. Initially, $V_0 = \{r\}$ and $\lambda_0(r) = \mathbf{m}_0$. Since $\mathbf{m}_0 \xrightarrow{\varepsilon} \mathbf{m}_0 = \lambda_0(r)$ the base case is established.
Assume that the assertions hold for $CT_n$. Observe that MinCov may change the labeling function $\lambda$ and/or add new vertices in exactly two places: at lines 4-6 and at lines 22-25. Therefore in order to prove the assertion, we show that after each group of lines it still holds.
• After lines 4-6: MinCov computes (1) a maximal fireable sequence $\sigma \in \mathsf{Acc}_n^*$ from $\lambda_n(u)$ (line 4), and updates $u$'s marking to $\mathbf{m}_u = \lambda_n(u) + \mathbf{C}(\sigma)$ (line 5). Since the assertions hold for $CT_n$, (2) if $u \neq r$, $\lambda_n(prd(u)) \xrightarrow{\delta(prd(u),u)} \lambda_n(u)$ else $\mathbf{m}_0 \xrightarrow{\delta(r,r)} \lambda_n(r)$. By concatenation, we get $\lambda_n(prd(u)) \xrightarrow{\delta(prd(u),u)\sigma} \mathbf{m}_u$ if $u \neq r$ and otherwise $\mathbf{m}_0 \xrightarrow{\delta(r,r)\sigma} \mathbf{m}_u$ which establishes that the assertions hold after line 6.
• After lines 22-25: The vertices for which $\lambda$ is updated at these lines are the children of $u$ that are added to the tree. For every fireable transition $t \in T$ from $\lambda(u)$, MinCov creates a child $v_t$ for $u$ (lines 22-23). The marking of any child $v_t$ is set to $\mathbf{m}_{n+1}(v) := \mathbf{m}_{n+1}(u) + \mathbf{C}(t)$ (line 24). Therefore since $\lambda_{n+1}(u) \xrightarrow{t} \lambda_{n+1}(v_t)$, the assertions hold.

■

**Lemma 2.** *At any execution point of MinCov,* $\mathsf{Acc}$ *is a set of accelerations.*

**Proof.** At most one acceleration is added per iteration. Let us prove by induction on the number $n$ of iterations of the main loop that $\mathsf{Acc}_n$ is a set of accelerations. Since $\mathsf{Acc}_0 = \emptyset$, the base case is straightforward.
Assume that $\mathsf{Acc}_n$ is a set of accelerations and consider $\mathsf{Acc}_{n+1}$. In an iteration, MinCov may add an $\omega$-transition $\mathbf{a}$ to $\mathsf{Acc}$. Due to the inductive hypothesis, $\delta(\gamma)$ is a sequence of abstractions where $\gamma$ is defined at line 9. Consider $b$, the $\omega$-transition defined by $\mathbf{Pre}(b) = \mathbf{Pre}(\delta(\gamma))$ and $\mathbf{C}(b) = \mathbf{C}(\delta(\gamma))$. Due to Proposition 2, $b$ is an abstraction. Due to Proposition 3, the loop of lines 11-15 transforms $b$ into an acceleration $\mathbf{a}$. Due to Proposition 7, after truncation at line 16, $\mathbf{a}$ is still an acceleration. ■

**Proposition 10.** $[\![\lambda(V)]\!] \subseteq Cover(\mathcal{N}, \mathbf{m}_0)$.

**Proof.** Let $v \in V$. Consider the path $u_0, \ldots, u_k$ of $CT$ from the root $r = u_0$ to $u_k = v$. Let $\sigma \in (T \cup \mathsf{Acc})^*$ denote $\delta(prd(u_0), u_0) \cdots \delta(prd(u_k), u_k)$. Due to Lemma 1, $m_0 \xrightarrow{\sigma} \lambda(v)$. Due to Lemma 2, $\sigma$ is a sequence of abstractions. Due to Proposition 2, the $\omega$-transition $\mathbf{a}$ defined by $\mathbf{Pre}(\mathbf{a}) = \mathbf{Pre}(\sigma)$ and $\mathbf{C}(\mathbf{a}) = \mathbf{C}(\sigma)$ is an abstraction. Due to Proposition 1, $[\![\lambda(v)]\!] \subseteq Cover(\mathcal{N}, \mathbf{m}_0)$. ■

The following definitions are related to an arbitrary execution point of M*inCov* and are introduced to establish its completeness.

**Definition 8.** *Let* $\sigma = \sigma_0 t_1 \sigma_1 \ldots t_k \sigma_k$ *with for all* $i$, $t_i \in T$ *and* $\sigma_i \in \mathsf{Acc}^*$. *Then the firing sequence* $\mathbf{m} \xrightarrow{\sigma} \mathbf{m}'$ *is an* exploring sequence *if:*

- *There exists* $v \in \mathsf{Front}$ *with* $\lambda(v) = \mathbf{m}$
- *For all* $0 \leq i \leq k$, *there does not exist* $v' \in V \setminus \mathsf{Front}$ *with* $\mathbf{m} + \mathbf{C}(\sigma_0 t_1 \sigma_1 \ldots t_i \sigma_i) \leq \lambda(v')$.

**Definition 9.** *Let* $\widehat{\mathbf{m}}$ *be a marking. Then* $\widehat{\mathbf{m}}$ *is* quasi-covered *if:*

- *either there exists* $v \in V \setminus \mathsf{Front}$ *with* $\lambda(v) \geq \widehat{\mathbf{m}}$;
- *or there exists an exploring sequence* $\mathbf{m} \xrightarrow{\sigma} \mathbf{m}' \geq \widehat{\mathbf{m}}$.

In order to prove completeness of the algorithm, we want to prove that at the beginning of every iteration, any $\mathbf{m} \in Cover(\mathcal{N}, \mathbf{m}_0)$ is quasi-covered. To establish this assertion, we introduce several lemmas showing that this assertion is preserved by some actions of the algorithm with some prerequisites. More precisely, Lemma 3 corresponds to the deletion of the current vertex, Lemma 4 to the discovery of an acceleration, Lemma 5 to the deletion of a subtree whose marking of the root is smaller than the marking of the current vertex and Lemma 6 to the creation of the children of the current vertex.

**Lemma 3.** *Let* $CT$, $\mathsf{Front}$ *and* $\mathsf{Acc}$ *be the values of corresponding variables at some execution point of MinCov and* $u \in V$ *be a leaf in* $CT$ *such that the following items hold:*

1. *All* $\mathbf{m} \in Cover(\mathcal{N}, \mathbf{m}_0)$ *are quasi-covered;*
2. $\lambda(V \setminus \mathsf{Front})$ *is an antichain;*
3. *For all* $\mathbf{a} \in \mathsf{Acc}$ *fireable from* $\lambda(u)$, $\lambda(u) = \lambda(u) + \mathbf{C}(\mathbf{a})$;
4. *There exists* $v \in V \setminus \{u\}$ *such that* $\lambda(v) \geq \lambda(u)$.

*Then all* $\mathbf{m} \in Cover(\mathcal{N}, \mathbf{m}_0)$ *are quasi-covered after performing* `Delete`$(u)$.

**Lemma 4.** *Let* $CT$, $\mathsf{Front}$ *and* $\mathsf{Acc}$ *be the values of corresponding variables at some execution point of MinCov. and* $u \in V$ *such that the following items hold:*

1. *All* $\mathbf{m} \in Cover(\mathcal{N}, \mathbf{m}_0)$ *are quasi-covered;*
2. $\lambda(V \setminus \mathsf{Front})$ *is an antichain;*
3. *For all* $v \in V \setminus \{r\}$, $\lambda(prd(v)) \xrightarrow{\delta(prd(v),v)} \lambda(v)$.

*Then all* $\mathbf{m} \in Cover(\mathcal{N}, \mathbf{m}_0)$ *are quasi-covered after performing* `Prune`$(u)$ *and then adding* $u$ *to* $\mathsf{Front}$.

**Lemma 5.** *Let* $CT$*,* Front *and* Acc *be the values of corresponding variables at some execution point of MinCov,* $u \in$ Front *and* $u' \in V$ *such that the following items hold:*

1. *All* $\mathbf{m} \in Cover(\mathcal{N}, \mathbf{m}_0)$ *are quasi-covered;*
2. $\lambda(V \setminus \mathsf{Front})$ *is an antichain;*
3. *For all* $v \in V \setminus \{r\}$*,* $\lambda(prd(v)) \xrightarrow{\delta(prd(v),v)} \lambda(v)$*;*
4. $\lambda(u') < \lambda(u)$ *and* $u$ *is not a descendant of* $u'$*.*

*Then after performing* `Prune`$(u')$; `Delete`$(u')$*,*

1. *All* $\mathbf{m} \in Cover(\mathcal{N}, \mathbf{m}_0)$ *are quasi-covered;*
2. $\lambda(V \setminus \mathsf{Front})$ *is an antichain;*
3. *For all* $v \in V \setminus \{r\}$*,* $\lambda(prd(v)) \xrightarrow{\delta(prd(v),v)} \lambda(v)$*.*

**Lemma 6.** *Let* $CT$*,* Front *and* Acc *be the values of corresponding variables at some execution point of MinCov. and* $u \in$ Front *such that the following items hold:*

1. *All* $\mathbf{m} \in Cover(\mathcal{N}, \mathbf{m}_0)$ *are quasi-covered;*
2. $\lambda(V \setminus \mathsf{Front}) \cup \{\lambda(u)\}$ *is an antichain;*
3. *For all* $\mathbf{a} \in$ Acc *fireable from* $\lambda(u)$*,* $\lambda(u) = \lambda(u) + \mathbf{C}(\mathbf{a})$*.*

*Then after removing* $u$ *from* Front *and for all* $t \in T$ *fireable from* $\lambda(u)$*, adding a child* $v_t$ *to* $u$ *in* Front *with marking of* $v_t$ *defined by* $\lambda_u(v_t) = \lambda(u) + \mathbf{C}(t)$*, all* $\mathbf{m} \in Cover(\mathcal{N}, \mathbf{m}_0)$ *are quasi-covered.*

**Proposition 11.** *At the beginning of every iteration, all* $\mathbf{m} \in Cover(\mathcal{N}, \mathbf{m}_0)$ *are quasi-covered.*

**Proof.** Let us prove by induction on the number of iterations that all $\mathbf{m} \in Cover(\mathcal{N}, \mathbf{m}_0)$ are quasi-covered.
Let us consider the base case. MinCov initializes $V$ and Front to $\{r\}$ and $\lambda(r)$ to $\mathbf{m}_0$. By definition, for all $\mathbf{m} \in Cov(\mathcal{N}, \mathbf{m}_0)$ there exists $\sigma = t_1 t_2 \cdots t_k \in T^*$ such that $\mathbf{m}_0 \xrightarrow{\sigma} \mathbf{m}' \geq \mathbf{m}$. Since $V \setminus \mathsf{Front} = \emptyset$, this firing sequence is an exploring sequence.
Assume that all $\mathbf{m} \in Cover(\mathcal{N}, \mathbf{m}_0)$ are quasi-covered at the beginning of some iteration. Let us examine what may happen during the iteration. In lines 4-6, MinCov computes the maximal fireable sequence $\sigma \in \mathsf{Acc}_n^*$ from $\lambda_n(u)$ (line 4) and sets $u$'s marking to $\mathbf{m}_u := \lambda_n(u) + \mathbf{C}(\sigma)$ (line 5). Afterwards, there are three possible cases: (1) either $\mathbf{m}_u$ is covered by some marking associated with a vertex out of Front, (2) either an acceleration is found, (3) or MinCov computes the successors of $u$ and removes $u$ from Front.

**Line 7.** MinCov calls `Delete`$(u)$. So $CT_{n+1}$ is obtained by deleting $u$. Moreover, $\lambda(u') \geq \mathbf{m}_u$. Let us check the hypotheses of Lemma 3. Assertion 1 follows from induction since (1) the only change in the data is the increasing of $\lambda(u)$ by firing some accelerations and (2) $u$ belongs to Front so cannot cover intermediate markings of exploring sequences. Assertion 2 follows from Proposition 9 since $V \setminus \mathsf{Front}$ is unchanged. Assertion 3 follows immediately from lines 4-6. Assertion 4 follows with $v = u'$. Thus using this lemma the induction is proved in this case.

**Lines 8-16.** Let us check the hypotheses of Lemma 4. Assertions 1 and 2 are established as in the previous case. Assertion 3 holds due to Lemma 1, and the fact that no edge has been added since the beginning of iteration. Thus using this lemma the induction is proved in this case.

**Lines 18-25.** We first show that the hypotheses of Lemma 6 hold before line 21. Let us denote the values of $CT$ and $\mathsf{Front}$ after line 20 by $\widehat{CT}_n$ and $\widehat{\mathsf{Front}}_n$. Observe that for all iteration of Line 19 in the inner loop, the hypotheses of Lemma 5 are satisfied. Therefore, in order to apply Lemma 6 it remains only to check assertions 2 and 3 of this lemma. Assertion 2 holds since (1) $\lambda(V \setminus \mathsf{Front})$ is an antichain, (2) due to Line 7 there is no $w \in V \setminus \mathsf{Front}$ such that $\lambda(w) \geq \lambda(u)$, and (3) by iteration of Line 19 all $w \in V \setminus \mathsf{Front}$ such that $\lambda(w) < \lambda(u)$ have been deleted. Assertion 3 holds due to Line 5 (all useful enabled accelerations have been fired) and Line 8 (no acceleration has been added).

Lines 21-25 correspond to the operations related to Lemma 6. Thus using this lemma, the induction is proved in this case.

■

The completeness of M*inCov* is an immediate consequence of the previous proposition.

**Corollary 1.** *When MinCov terminates,* $Cover(\mathcal{N}, \mathbf{m}_0) \subseteq [\![\lambda(V)]\!]$.

**Proof.** By Proposition 11 all $\mathbf{m} \in Cover(\mathcal{N}, \mathbf{m}_0)$ are quasi-covered. Since on termination, $\mathsf{Front}$ is empty for all $\mathbf{m} \in Cover(\mathcal{N}, \mathbf{m}_0)$, there exists $v \in V$ such that $\mathbf{m} \leq \lambda(v)$. ■

### 4 Tool and benchmarks

In order to empirically evaluate our algorithm, we have implemented a prototype tool which computes the clover and solves the coverability problem. This tool is developed in the programming language Python, using the Numpy library. It can be found on GitHub[3]. All benchmarks were performed on a computer equipped by Intel i5-8250U CPU with 4 cores, 16GB of memory and Ubuntu Linux 18.03.

**Minimal coverability set.** We compare `MinCov` with the tool `MP` [14], the tool `VH` [16], and the tool `CovProc` [10]. We have also implemented the (incomplete) minimal coverability tree algorithm denoted by `AF` in order to measure the additional memory needed for the (complete) tools. Both `MP` and `VH` tools were sent to us by the courtesy of the authors. The tool `MP` has an implementation

[3] https://github.com/IgorKhm/MinCov in Python and another in C++. For comparison we selected the Python one to avoid biases due to programming language.

We ran two kinds of benchmarks: (1) 123 standard benchmarks from the literature in Table 1, (which were taken from [2]), (2) 100 randomly generated Petri nets also in Table 1, since the benchmarks from the literature do not present all the features that lead to infinite state systems. These random Petri nets have the following properties: (1) $50 < |P|, |T| < 100$, (2) the number of places connected of each transition is bounded by 10, and (3) they are not structurally bounded. The execution time of the tools was limited to 900 seconds.

Table 1 contains a summary of all the instances of the benchmarks. The first column shows the number of instances on which the tool timed out. The time column consists of the total time on instances that did not time out plus 900 seconds for any instance that led to a time out. The #Nodes column consists of the peak number of nodes in instances that did not time out on any of the tools (except `CovProc` which does not provide this number). For `MinCov` we take the peak number of nodes plus accelerations. In the benchmarks from the literature

**Table 1.** Benchmarks for clover

| **123 benchmarks from the literature** | | | | **100 random benchmarks** | | | |
|---|---|---|---|---|---|---|---|
| | T/O | Time | #Nodes | | T/O | Time | #Nodes |
| `MinCov` | 16 | 18127 | **48218** | `MinCov` | **14** | 13989 | **61164** |
| `VH` | **15** | **14873** | 75225 | `VH` | 15 | **13692** | 208134 |
| `MP` | 24 | 23904 | 478681 | `MP` | 21 | 21726 | 755129 |
| `CovProc` | 49 | 47081 | N/A | `CovProc` | 80 | 74767 | N/A |
| `AF` | 19 | 19223 | 45660 | `AF` | 16 | 15888 | 63275 |

we observed that the instances that timed out from `MinCov` are included in those of `AF` and `MP`. However there were instances the timed out on `VH` but did not time out on `MinCov` and vice versa. `MinCov` is the second fastest tool, and compared to `VH` it is 1.2 times slower. A possible explanation would be that `VH` is implemented in C++. As could be expected, w.r.t. memory requirements `MinCov` has the least number of nodes. In the benchmarks from the literature `MinCov` has approximately 10 times less nodes then `MP` and 1.6 times less then `VH`. In the random benchmarks these ratio are significantly higher.

**Coverability.** We compare `MinCov` to the tool `qCover` [2] on the set of benchmarks from the literature in Table 2. In [2], `qCover` is compared to the most competitive tools for coverability and achieves a score of 142 solved instances while the second best tool achieves a score of 122. We split the results into safe instances (not coverable) and unsafe ones (coverable). In both categories we counted the number of instances on which the tools failed (columns T/O) and the total time (columns Time) as in Table 1.

We observed that the tools are complementary, i.e. `qCover` is faster at proving that an instance is safe and `MinCov` is faster at proving that an instance is unsafe.

**Table 2.** Benchmarks for the coverability problem (60 unsafe and 115 safe)

| | Time Unsafe | T/O Unsafe | Time safe | T/O safe | T/O | Time |
|---|---|---|---|---|---|---|
| `MinCov` | **1754** | **1** | 51323 | 53 | 54 | 53077 |
| `qCover` | 26467 | 26 | **11865** | **11** | 37 | 38332 |
| `MinCov` ∥ `qCover` | 1841 | 2 | 13493 | 11 | **13** | **15334** |

Therefore, by splitting the processing time between them we get better results. The third row of Table 2 represents a parallel execution of the tools, where the time for each instance is computed as follows:

$$\text{Time}(\texttt{MinCov} \parallel \texttt{qCover}) = 2 \min\left(\text{Time}(\texttt{MinCov}), \text{Time}(\texttt{qCover})\right).$$

Combining both tools is 2.5 times faster than `qCover` and 3.5 times faster than `MinCov`. This confirms the above statement. We could still get better results by dynamically deciding which ratio of CPU to share between the tools depending on some predicted status of the instance.

### 5 Conclusion

We have proposed a simple and efficient modification of the incomplete minimal coverability tree algorithm for building the clover of a net. Our algorithm is based on the introduction of the concepts of covering abstractions and accelerations. Compared to the alternative algorithms previously designed, we have theoretically bounded the size of the additional space. Furthermore we have implemented a prototype which is already very competitive.

From a theoretical point of view, we plan to study how abstractions and accelerations, could be defined in the more general context of well structured transition systems. From an experimental point of view, we will follow three directions in order to increase the performance of our tool. First as in [13], we have to select appropriate data structures to minimize the number of comparisons between $\omega$-markings. Then we want to precompute a set of accelerations using linear programming as the correctness of the algorithm is preserved and the efficiency could be significantly improved. Last we want to take advantage of parallelism in a more general way than simultaneously running several tools.
